# A student‐led qualitative study to explore dental undergraduates' understanding, experiences, and responses to racism in a dental school

**DOI:** 10.1111/jphd.12514

**Published:** 2022-06-21

**Authors:** Arefeh Ahmadifard, Sara Forouhi, Paula Waterhouse, Vanessa Muirhead

**Affiliations:** ^1^ Institute of Dentistry, Faculty of Medicine and Dentistry Queen Mary University of London London UK; ^2^ Newcastle University Faculty of Medical Sciences Newcastle upon Tyne UK

**Keywords:** dental, intersectionality, power, professional, racism, schools, sexism, students

## Abstract

**Objectives:**

This qualitative study explored dental student participants' understanding of racism, their experiences, and responses to racism in dental school, and the impacts of their experiences.

**Methods:**

An interpretative phenomenological analysis design recruited students from the undergraduate dental degree and the BSc in Oral Health Science course at a UK dental school in December 2020. Two students and a qualitative researcher facilitated the online focus groups. A topic guide including scenario questions guided the discussions that lasted an average of 2 h. The recorded interviews were transcribed and analyzed using thematic analysis.

**Results:**

Twenty‐five participants took part in five focus groups. Several themes emerged related to participants' experiences and reactions. They described a spectrum of racist encounters ranging from more subtle forms, such as stereotyping and microaggression to racial mocking. They were concerned about professionalism, not knowing how and when to respond to patients' racist behavior. They described gender discrimination and intersectional biases but felt compelled to put patients' interests first. They were unsure about how to respond to stereotyping or racism from staff because of perceived imbalances in the staff‐patient‐student triad relationship. They expressed fears of gaslighting and despondency. They also felt that the COVID‐19 pandemic and anonymity from virtual learning environments enabled racist behavior.

**Conclusion:**

This study revealed a complex triad relationship between participants, staff and patients, and experience of intersectionality and three levels of racism: interpersonal, structural, and institutional. It highlighted the need for further research to develop actions including structural policies and equality and diversity training.

## INTRODUCTION

Racism can be defined as the beliefs and/or attitudes about the perceived inferiority of a particular race and ethnicity leading to racial discrimination and unequal treatment based on race and ethnicity [1]. Racism encompasses behaviors that range from physical or verbal abuse to subtle and covert manifestations such as microaggression [2]. Sociologists have also recognized that racism does not only encompass overt behaviors but also covert, unconscious or implicit biases and stereotyping [1].

The dynamic nature of racial discourse has led to expanded definitions, acknowledging that racism can exist and be experienced at different levels. Interpersonal racism operates at the micro‐level or person‐to‐person level, arising from individual prejudices and beliefs [3]. Structural racism occurs when racism permeates into social policies and structures [4]. Structural racism (used synonymously with systemic racism) describes how this form of racism persists in cultural, social, economic, and environmental milieux to reinforce racial inequities. Institutional racism refers to the further pervasiveness of racism, operating at the macro‐level in the policies, practices, and cultures of an institution that establish racial hierarchies and perpetuate inequalities [5, 6]. One example of this would be differential actions in complaints procedures, recruitment, and promotion within an organization [7]. The complex and endemic nature of racism is in part fuelled by the fact that these different forms do not exist in isolation but are closely interlinked. For example, interpersonal racist behaviors may be enabled and normalized by the culture of an organization established through institutional racism [8].

Previous research has described the psychological, social, and educational impacts of primarily interpersonal racism encountered by students, including anxiety and depression, social isolation, low self‐esteem, and decreased academic performance [9, 10]. Racial discrimination experienced by ethnic minority university students also influences their access to mental health support services [11].

In 2020, the murder of George Floyd and the associated *Black Lives Matter* movement generated a public discourse on racism with renewed focus [12]. This stimulated multiple institutions and health professional students to report their lived experiences of racial injustice and discrimination [13, 14]. Research from medical schools showed that only half of UK medical schools collect data on students' complaints about racism and racial harassment [15]. Furthermore, students from ethnic minority backgrounds who experience racism are often dissuaded from reporting racial incidents, reflected in the very low number of complaints [15, 16]. Anecdotal reports in Dentistry suggest that racism prevails in various forms from verbal or physical abuse to microaggression and unfair treatment due to ethnicity, skin color, accent or religion, but such experiences have not been systematically studied [17]. Beyond these reports, we know very little about the experience of interpersonal, structural, and institutional racism in dental school because of the paucity of research about the lived experiences of dental students, who not only interact with tutors but with other students, patients, with academic institutions and the wider dental profession. Identifying evidence of structural and institutional racism in dental school would also raise questions about the institutional duty of care to students from ethnic minority backgrounds when racism is experienced yet students may be dissuaded from making complaints.

This qualitative study aimed to gain a deeper understanding of the perceptions and experiences of dental students or racism, discrimination, and inequality in a UK dental school. We also explored the impacts of racism on dental students and students' responses to racism to identify any barriers that hinder effectively reporting or dealing with racial harassment and discrimination. Understanding dental students' awareness, experiences, and responses to interpersonal, structural, and institutional racism in dental school is an essential step in developing effective training opportunities, resources, and policies to tackle racism.

## METHODS

### Participants

The participants were undergraduate students currently enrolled in any year of the 5‐year Bachelor of Dental Surgery (BDS) degree and the Bachelor in Oral Health Sciences (BSc) 3‐year programmes at the Institute of Dentistry, Queen Mary University of London (QMUL), UK. There were no restrictions placed on age, gender, or ethnicity of the participants. This dental school is a teaching hospital situated in East London, UK in a multi‐ethnic, socially deprived area, which serves a diverse range of patients. Its students (under supervision by clinical tutors) provide comprehensive free dental treatment to patients. Sixty‐six percent of the dental student cohort is female. The dental students come from diverse ethnic backgrounds (Asian 67%; White 13%, Arab 7%, Black 6%, Mixed ethnicity 3% and, other 3%). Sixty percent of academic staff is White, 28% Asian, 4% Black and 9% from other ethnic groups. Fifty percent of academic staff is female.

### Study design and theoretical perspective

This qualitative study used an Interpretative Phenomenological Analysis (IPA) study design to explore dental students' perceptions and experiences of racism in the dental school environment [18]. IPA was chosen because of its previous utility in racial equality education research [19], allowing the students to integrate their beliefs and memories across their student journeys [20]. Using IPA enabled the students to appreciate and to reflect on their own unique voices and the shared experiences of their fellow students.

We used a critical counter‐narrative theoretical framework: a transformative methodology that adopts Critical Race Theory (CRT), counter‐narratives and counter‐storytelling [21]. CRT considers racism to be pervasive and endemic, influencing all aspects of society including academia, challenging notions of neutrality and apolitical praxis [22, 23]. A critical counter‐narrative theoretical perspective also views research, policies and practices within their contemporary and cultural context, discovering meaning from an intersectionality lens of multiple intersecting identities and concurrent oppression and privilege [24]. Counter‐storytelling emphasizes the narratives of students perceived as underprivileged compared to the dominant culture [25]. The dominant culture describes the ideas and beliefs of the institutions (e.g., university) and the structures that operate in these institutions and dictated by dental professional organizations such as the General Dental Council (GDC) that regulates dental teaching and training in the United Kingdom. The dental school academic structure also makes patients and tutors more power dominant relative to dental students [25].

The Queen Mary University of London Research Ethics Committee gave ethical approval for the study on the 14 October 2020 (QMERC2020/48).

### Sampling and recruitment

Maximum variation purposive sampling was used to recruit participants who were undergraduate dental students from across the study body. The aim of maximum variation (or heterogeneous) sampling is to select a wide range of participants from typical to extreme cases, to identify common themes and to gain broader insights and varying perspectives [26]. All students were invited to participate regardless of their gender, race, and ethnicity and whether they had personally experienced racism at the dental school. This was consistent with the maximum variation sampling strategy and the aim to explore interpersonal, structural, and institutional racism.

The two dental student researchers led the recruitment using a range of approaches including a formal email invitation sent out to the students by the Director of Undergraduate Studies, recruitment posters and social media posts on dental society web pages. Sample size requirements for qualitative research are not based on sample size calculations but on thematic saturation [27]. Data gathering and analysis were concurrent; recruitment and data analysis continued until no new themes emerged from the focus groups.

### Data collection

The participants who agreed to take part completed an online background questionnaire to collect information about their age, gender, race and ethnicity, year, and course of study. Two final‐year dental student researchers (AA, SF) and an experienced qualitative researcher (VM) jointly facilitated the semi‐structured focus group interviews, recorded on Microsoft Teams. The student researchers were trained to lead the initial questioning with further probes and prompts provided by the experienced qualitative researcher. Topic guided focus groups were piloted on a group of students before the focus groups. The topic guide was developed from a review of the literature and from stories shared by students [28, 29]. These stories were used to develop the exploratory hypothetical scenarios in “Introduction” section of the topic guide, presenting four hypothetical scenarios illustrating encounters between students, tutors, and patients. These were designed to encourage discussion about participants' understanding and reflections on racist incidents (Appendix S1). Further prompts and probes explored their understanding of key definitions including racism, unconscious bias, and privilege. “Methods” section delved into their personal experiences of racism in the dental school. The final two sections explored their responses to racism and the impacts that racism had on them as victims or witnesses. We grouped the participants into year groups; the number of participants in each focus group ranged from four to seven students. The focus groups lasted between 101 and 125 min (total = 11 h). Each participant received a £20 Amazon voucher for participating.

### Data analysis

The audio‐recordings from the online focus groups were transcribed using intelligent transcript and analyzed using a six‐step Thematic Analysis process [30]: (1) familiarization, (2) generating codes, (3) searching for themes, (4) reviewing the themes, (5) defining and naming the themes and (6) writing up the findings. The three coders (AA, SF, VM) initially read the transcripts to search for meanings and patterns (step 1). The initial codes were generated to organize the data into meaningful groups using line‐by‐line coding and Microsoft Word software (AA, SF, VM) (step 2). Exhaustive coding generated a list of different codes that were sorted and analyzed to search for full‐realized themes (step 3). We reviewed and refined the themes by re‐reading the excerpts to confirm their coherence (step 4) and named the themes (step 5). The final stage was identifying the counter‐narratives to illustrate the themes (step 6).

The research team consisted of two final‐year dental students (AA, SF), a qualitative researcher and Dental Public Health specialist (VM) and a Pediatric Dentistry academic and former Lead of Equality, Diversity, and Inclusion in a dental school (PW). The research team was all female. The two student researchers were from a British Asian (Iranian) and British multiethnic Asian (Indian and Iranian) ethnic background. The two staff members were from a White British and Black British ethnic background. We established rigor and trustworthiness by member checking, and sharing the findings with the Anti‐Racism Steering Group Committee [31] and with the wider staff and student steering group. We ensured dependability through bracketing before and after the focus groups and in the debriefing, to expose our presuppositions and biases throughout the research process and during the data analysis carried out by multiple coders [32].

## RESULTS

Five focus groups were conducted with 25 participants. The sample included first‐year BSc students and students from all 5 years of the BDS course. Seventy‐two percent of the participants were female and 72% were Asian, which reflected the ethnic distribution of the students at the dental school. Two participants (8%) were White, one participant was Black (4%), and one participant was of mixed ethnicity (4%) (Table 1).

**TABLE 1:** Sociodemographic and study characteristics of the dental students who participated in the focus groups (*N* = 25)

Characteristics	Number (%)
Gender	
Female	18 (72%)
Male	7 (28%)
Year group	
Year 1 BSc in Oral Health Sciences students	3 (12%)
Year 1 undergraduate (BDS) dental students	2 (8%)
Year 2 undergraduate (BDS) dental students	4 (16%)
Year 3 undergraduate (BDS) dental students	4 (16%)
Year 4 undergraduate (BDS) dental students	5 (20%)
Year 5 undergraduate (BDS) dental students	7 (28%)
Ethnic groups	
Asian	18 (72%)
Black	1 (4%)
White	2 (8%)
Mixed	1 (4%)
Other	3 (12%)

Abbreviations: BDS, Bachelor of Dental Surgery; BSc, Bachelor of Sciences.

**FIGURE 1:**
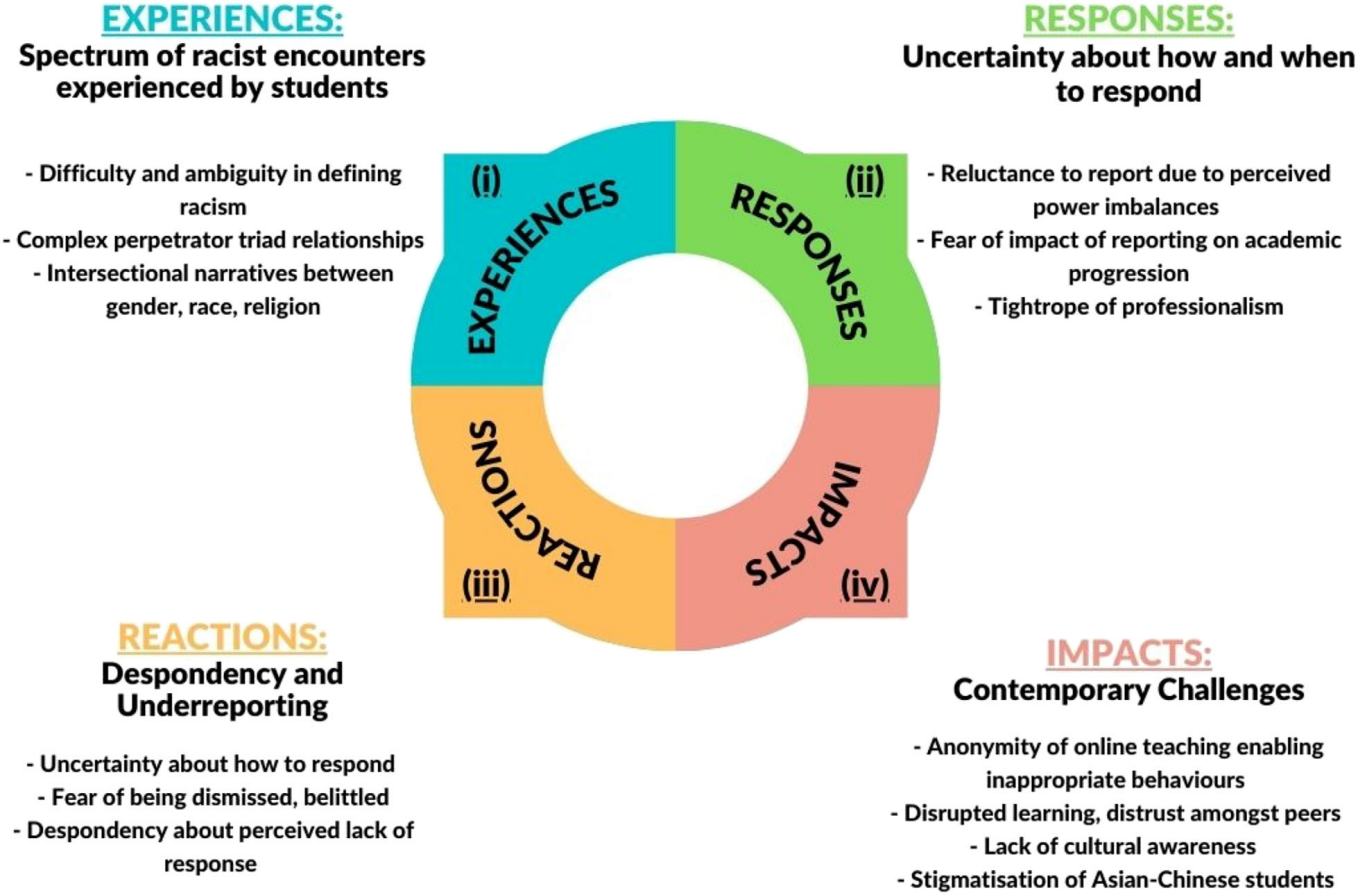
Themes generated from the qualitative study exploring dental students' experiences of racism in a dental school [Color figure can be viewed at wileyonlinelibrary.com]

Four main themes emerged from the focus groups, as depicted in Figure 1 and described below with illustrative counter‐narratives: (i) Experiences: the spectrum of racist encounters experienced by the participants (ii) Responses: Uncertainty about how and when to respond to patients' and tutors' racist behaviors; (iii) Reactions leading to underreporting or ignoring issues and despondency; and (iv) Impacts—the impact of contemporary challenges on participants' experiences of racism in the dental school.

### Experiences: The spectrum of racist encounters experienced by the participants

The scenarios in the topic guide triggered the participants' own recollections of a range of incidents that they had personally experienced or witnessed at the dental school. The incidents on this spectrum varied from more subtle or covert forms including racial stereotyping, racial microaggression and racial mocking, to overt forms of racial encounters. Examples of microaggression included tutors not remembering the participants' names and patients complimenting students on their English‐speaking ability. These incidents had involved participants, tutors, and patients. The participants expressed some uncertainty about whether some of the more subtle occurrences had racist undertones. They described how they had often shared their experiences with close friends and family who reassured them that the incidents were inappropriate. Table 2 shows the counter‐narratives that describe this spectrum.

**TABLE 2:** Counter‐narratives that depict the spectrum of racist and intersectional discriminatory encounters that dental students had experienced in the dental school

Direct quotations from the focus group transcripts			
Racial stereotyping	Racial microaggressions	Racial mocking	Overt racist behaviors
“One of my experiences was on one of our outreach clinics and we had finished doing whatever treatment that we were doing and me and the other student had finished. And then the patient was like, “Oh, it's really nice that this school picks people from poor disadvantaged areas to become dentists.” In the beginning, I don't think we really understood what had just happened. But then at the end, when I was kind of thinking back, I was like wait, what is this woman just saying. And yeah, I was kind of in shock, but I just left it there because I just didn't really know what to say with that kind of statement. I think I just kind of looking back on it, I don't understand why someone would make such a statement. I wanted to be sure that would even happen in the clinic, because I am Black, and the other student was Muslim. I think she made it sound like it was… they were doing some sort of charity scheme rather than you getting in based on your merits, it's like they let you in because they wanted to help disadvantaged people and, yeah, you'd sort of question if they even thought you were capable, or… yeah, that's a really awkward and uncomfortable situation to be in.” (Focus group 5)	“I experienced microaggression in the sense that like I'm Asian and I've learnt English, it was my first language. I speak English like my whole life, but I get complemented when people say, “Oh your English is so good.” Um, like where are you from? Or so you speak perfect English, but English was the language I grew up speaking so. I think that's an example of microaggression.” (Focus group 2)	“This tutor randomly went up to me and said in front of my patient, “Do you like milkshake” and I was like no, not really. Why and then he went. “You look like the type of person to throw a milkshake at Nigel Farage.” [Former Leader of the UK Independent Party}. And I just was like, I think it had recently happened when somebody threw a milkshake at Nigel Farage. And then I went to the tutor. I said, “Oh is that because I'm Brown” and they were just like “Oh no no, I didn't. I didn't mean that I was just joking.” I just sort of stopped talking to them. I just sort of turned and just carried on doing what I was doing. And after that incident, that tutor never spoke to me again for a whole of [dental specialty] rotations. I was with him for the summer term and for that whole summertime, they did not speak to me once. They would even struggle actually to look at me. It was actually quite awkward. But it was just such a bizarre thing to happen. It was so random, and I was like “what”. I didn't find it funny. I found it really inappropriate. And I was actually just quite shocked it happened because I was in 3rd year at the time.” (Focus group 4)	“The [lecturer] was using mentimeter and there were, I think three students who put their names as racial slurs. It was noticed by people instantly who I think they got in touch with [lecturer], but the session just carried on. Nothing was initially said the following session. They noted it at the beginning, and they said, “Look this happened last time; it will not be tolerated.” We know who you are, and someone did it again. And everyone noticed, everyone mentioned it, and for especially [student's name] felt the same. It was almost impossible to concentrate knowing that had happened and that people were concerned about it, and nothing was being said.” (Focus group 1)
	“And a few times, it's a similar situation to one of the examples that you looked at. Some tutors don't make the effort to remember names, so a few times I have been called the name of other people in my group [name of student] simply because they have said “your pretty much known as [name] anyway because you're a Muslim.” That has happened quite a few times. I forgot which tutors they were and it's probably best not to mention them anyway. That happened earlier on. (Focus group 4)		I think the other day when I was walking, while I'm walking back from dental school and then some random guy just came up to me and said like, “Oh, you Hong Kong [expletive].” And I was like what? I'm not from Hong Kong but OK whatever. And yeah, I just walked past, but I felt like that was quite racist just because. he just assumed that I was from Hong Kong based on my skin colour. Then he just called me {expletive] just because he thought I was from Hong Kong.” (Focus group 4)
**Experiences of intersectional discrimination**			
“I had that experience; it was at [location] with a female patient. She requested a female practitioner. At the time, I was very new to dentistry. I was, I just assumed it was like an OK, a normal preference. And myself, I personally didn't have any qualms about it, so I was happy to offer. But then upon reflection, and you know speaking to my tutors, I sort of realised that you know, when patients agree to treatment, they can have preference. They can have reasonable preferences. But when it comes to dental treatment, it not necessarily a gendered procedure. It does not need a gendered professional. I understand because my tutor was adamantly against it, but we eventually did. We did comply with the patient's wishes. I think there were plenty of students to see her. But yeah, I think the principle behind it didn't quite make sense upon reflection.” (Focus group 4)			
“We were also doing oral surgery with three males. There was just one female who was my clinical partner, so when they [patient] asked if it was possible just to be in an area with females, then we're all pretty shocked with that. Because they were obviously conservative and wanted to be quite religious. So, then we explained to them that we could do that, but it would be asking a lot of everyone in the entire clinic to leave just for this one patient if they really wanted to just have a female area. I turned around and the husband was like you can't be here. So, I felt pretty pretty shocked, just to hear that because I was. I wasn't even doing anything. I was looking away.” (Focus group 4)			

Some participants described not only racial incidents but also experienced and/or witnessed gender discrimination from patients who specifically wanted a dental student from a particular gender to treat them (Table 2). The participants also described other intersectional discrimination from patients based on their religion, most commonly Islamophobia (Table 2).

### Responses: Uncertainty about how and when to respond to patients and tutors' racist behaviors

The degree of uncertainty about their racist encounters and the ambiguity about how to define racism meant that the participants were often unsure about how to respond to patients' remarks. They reflected on their training on professionalism and the first principle of the General Dental Council (GDC) to “Put patients' interests first” [33]. They described their concerns about the tension between being professional and confronting patients' racist behaviors. This tight rope of professionalism or desire to be professional often meant that they ignored or dismissed patients remarks as illustrated in the counter‐narratives in Table 3.

**TABLE 3:** Counter‐narratives to illustrate students' responses to patients' and tutors' racist behaviours at the dental school

Direct quotations from focus group transcripts
“You're always told that patients are the priority. Everything we learn about patients; the patient comes first and then they're showing a blatant disregard and no decency. And although there are some things you should overlook when you know you're treating a patient, some awkward remarks. You just kind of think, “No; the patient care comes first. Sometimes we do not confront these sort of things and say, “listen, this isn't acceptable. I'm here to abide by certain set rules. You agreed to be treated in this environment.” (Focus group 3)
“I say that the patient is in a position of power because if you're treating a patient and you're acting as a professional and I guess like what I would probably do is treat a patient in a professional manner which means that you still follow the GDC {General Dental Council] principles, you still have to put the patient's interest first so does it really matter that the patient likes or doesn't like you? But you have a duty to care and if that means putting your patient out of pain and that's what you do, you put a patient out of pain.” (Focus group 4)
“I had one experience in [Location] last year. I had a patient who we got on really well with each other. He was really nice every time he visited, he'd bring me chocolate and presents. And then once we were talking about holidays and for some reason, I mentioned Turkey and he said, “Oh, I don't like Turkey; too many Muslims there”. So that was really shocking because obviously this comment was unacceptable because why would you not like a place because there's too many Muslims? I didn't think he realised that I was, I am Muslim, and I just didn't know how to respond, especially because of the fact that we got on well. So again, it was something that I would just brush under the carpet, and we moved on. But I did mention it to my clinical group later and I think someone in my group then mentioned it to a tutor on a different occasion. And then the tutor came to me and spoke to me that day and said, “Are you OK?” And to be honest like I was OK, I wasn't particularly offended. I was just more shocked because I didn't respond.” (Focus group 5)
“It's just still very new to clinic and how this sort of patient‐tutor environment and how the dynamics work. And it definitely feels perhaps hard, you know, to be able to call out different tutors at different times. I understand that tutors are people too. You know, they are people, but at the time it can sometimes feel like, I think the power differential is still very apparent. Definitely, I can empathise with that part of the story, you know. Third year is probably the hardest.” (Focus group 4)

The participants also described being reluctant to challenge tutors because of the perceived power imbalance between students and tutors. Some participants had confronted tutors and described how this had adversely affected their relationship with the tutors. Others described their fears and concerns about the ramification of confronting tutors and the likely impact that this could have on their academic progression (Table 3). Some participants delayed responding to tutors before having their concerns reaffirmed by their peers; they felt that the delay meant any resulting action was then inappropriate and ill‐timed.

### Reactions: Uncertainty leading to underreporting or ignoring issues and despondency

The uncertainty about how to respond to racist encounters meant that many participants decided not to report them. Some participants feared accusations of overreacting and being penalized for raising an issue. Other participants described tutors dismissing their previous reports; they felt belittled, leading to feelings of discouragement to report further incidents. The participants also described feeling despondent about the perceived lack of response or action to previous reports as described in the counter‐narratives in Table 4.

**TABLE 4:** Counter‐narratives to illustrate students' reactions to racist incidents at the dental school

Direct quotations from focus group transcripts
“I probably would have gone to someone else or complained or reacted differently, but I feel as though there's no point in making a scene because nothing happens. So even if I were to complain, and I don't want to be like controversial here, but there's nothing that can be done. All the tutors tend to be quite chummy with each other and that kind of attitude, whereas you know, I've mentioned it, I have complained about things in the Institute in the past, not because of racism because of other things in general. The response is “Oh, life is unfair” or stuff like that so then I realize that I'm going to be here for five years. There's no point in making a big fuss about it. I just have to get used to it, I guess over time.” (Focus group 4)
“To answer your questions, again, we've talked about, again, fear of backlash and then just lack of information. I think the other one we've touched on is how serious is it not wanting to make waves. It's almost as if it's, sort of, endemic if it's sort of normalised. Am I the one who's making a fuss? Then again, maybe there needs to be more effort to say “Actually, no, hold up. This isn't okay. So just I think that that's a good step, counteract that, but yeah, so another barrier if it's been normalised or if you feel it's not severe enough or if you feel like there needs to be a certain level.” (Focus group 2)
“There was like an incident, like, a while back where we had, like, online lectures and then we went into breakout groups and apparently someone said, like, racial and homophobic slurs. And people complained about it, obviously. And it was brought to everyone's attention. I guess, it's just concerning. Because, like, we have a duty as healthcare providers to be unbiased and to be unprejudiced and give everyone the treatment they deserve, for people to use these slurs in such a way makes me feel like they do have negative opinions of these people. And it just makes me question, like, can you really give proper treatment to treat everyone equally if you do you have these views?” I guess so, like, because obviously, like, no one knows who said it. It's a bit unnerving, I guess, because you don't exactly know who has these views.” (Focus group 3)
“Yes, I was just going to say, I think it would definitely be the right thing to do but just imagining myself in {Student's] position, I can imagine it being quite difficult because obviously you want a good relationship with the patient and then you don't want to ruin that and sort of create that divide that you went to a tutor and you brought it up and they had to be spoken to. You'd hope that they'd understand and maybe because they seem quite nice that they would understand but you'd hope it doesn't affect that relationship. I would say the tutors on clinics are generally really good, but a potential barrier would be if the tutor doesn't take it seriously or doesn't really feel the need to do anything to try to rectify the situation. Yeah, I feel like in situations like that normally how you respond is based on how secure you feel in your tutor to support you. If you feel sort of secure in your tutor, I think you are more likely to talk to them, I think you are more likely to come out of the situation even not feeling as a victim or not feeling in any bad way at all. I think it's just nice to have support because you know that in any way you will not be looked bad at this situation that happened to you.” (Focus group 5)

### Impacts: Contemporary challenges influencing participants' experiences of racism

The focus groups were carried out during a time of significant disruption caused by the COVID‐19 pandemic. This contemporary challenge created unprecedented impacts that were reflected in the participants' narratives. One impact was related to the shift from face‐to‐face to online teaching, which created a level of anonymity that fostered inappropriate online behaviors. An example was an online seminar, where the participants recalled their reactions when a student had anonymously posted racial and homophobic slurs on an online interactive platform. They described feeling shocked and distracted during the session. This led to long‐term feelings of distrust among the participants because no one knew who had posted the derogatory remarks (Table 5).

**TABLE 5:** Counter‐narratives to illustrate the impacts that racist incidents in the dental school had on dental students

Direct quotations from the focus group transcripts
“I think that the other thing was as well think. I don't know if anyone else noticed that when he was on it, but someone put a name. I think it was like [homophobic slur] or something, but something that's really silly and like really immature and it was addressed then and there, “Be sensible, you're here to learn. You've earned your place on this course. Use it.” But something that was so much bigger wasn't addressed right away. So that was disappointing. Or was it shocking? Yeah, I think it just took me back a bit. I was like OK, well, you say that's silly, which OK. But then something that is going to impact people potentially psychologically should be addressed straight away? I mean OK. It was addressed in the way it was dealt with, which was good, but it should have been addressed right away instead.” (Focus group 1)
“With Covid, you're trying to minimise treatments that require aerosol generating procedures, and when doing so, you have to wear masks. They filter out the air, but they have to be fitted to your skin and if you've got a beard your seal around, it interrupts the seal, so it isn't complete. And to basically to keep a beard and to do an AGP procedure, you have a hood that separates your head from the rest of your body. It's a hood that you have to wear and apparently the cost of it is £800. I don't know how true that is personally, but I know for a fact from I have quite a few friends in other universities around the country. I've got friends in Cardiff, Manchester, Birmingham, Sheffield, Kings, and we're like one of the only unis which hasn't had this setup for people with beards. It got to the point where they were telling us “OK, now you're going to have to shave if you want to see patients” and then people had to put their foot down. I don't know if I'd say it's racism. I think it's just a lack of acceptance. I think it's also a matter of ignorance because one of the tutors (I am not naming names), one of the tutors potentially did some research. I don't know how well they searched it, but they said that some scholars said that you don't have to keep a beard.” (Focus group 3).
“I didn't think this was racist or weird until I was talking to someone about it recently, and they said that it is not really something appropriate that they should've said, and it happened on clinic as well. And this was like last year I think at the end of last year and I had a tutor. And just after the huddle, he turned to me and he said, “Have you been coughing recently?” And I was like (this is like just when COVID started) and he was like “where have you been recently?” He just asked me like where have you been? And then I was like, I knew I haven't been to China right because of the current coronavirus. And at that time, he said that it was just a joke; he's just messing around. But that's not a very nice comment to make.” (Focus group 2)

Infection Control guidelines for COVID‐19 also meant that the participants had to be allocated Covid‐specific Personal Protective Equipment (PPE) to allow them to safely treat patients. The participants relayed the experiences of some students who were not able to wear standard facemasks because they chose to have beards for religious reasons. Some students felt that there was ignorance and a lack of cultural awareness and acceptance when these students were asked to shave their beards to see patients (Table 5). An Asian‐Chinese participant reported feeling stigmatized in the early phases of the COVID‐19 pandemic when they received racist and other inappropriate comments and questioning based on their race and ethnicity (Table 5).

## DISCUSSION

This is (to the authors' knowledge), the first study exploring dental students' understanding, experiences, responses, and reactions to racism in dental school. It uncovered several novel findings. The participants described a spectrum of interpersonal racism (including stereotyping and microaggression) in their encounters with other students, tutors, and patients. Microaggressions are every day, subtle, intentional, or unintentional communications or behaviors that convey bias towards a marginalized group [34]. The participants' experiences of these more subtle and hidden forms of interpersonal racism is consistent with previous studies in healthcare learning environments [35]. Although these covert forms of interpersonal racism may be unintentional and unconscious, they are often more insidious, causing individuals to question or doubt their experiences [36]. Studies have also shown the cumulative effects of microaggression negatively affecting students' mental health and learning [34, 37]. The participants often delayed reporting this covert but more common microaggression and stereotyping encounters, unsure if they had been subject to discrimination. The doubt associated with defining these covert racial encounters might explain why racial encounters in dental schools could be possibly under‐reported, with participants only reporting their more overt racial encounters.

The participants' descriptions of how they responded to racist remarks by patients, showed how they tried to balance their professionalism and duty of care to patients, seen as a barrier to responding to discriminatory behavior and interpersonal racism. This prioritization of patients raises the question about whether professional standards such as “putting patients interests first” [33] could enforce structural racism, where the structure is actually the dental profession. Moreover, is the “putting patients interests first” ideology a demonstration of how rhetorical power in professionalism could be used to the detriment of students who are subjected to interpersonal racism? [38]. Professionalism is an ideological discourse, deeply embedded in the culture of dentistry, which could perpetuate a divisive tension for dental students who will be future dentists [39]. Professionalism automatically sets up a hierarchy in which the voices of dental students are diminished to prioritize patients. This allows interpersonal racism to go unchallenged. Both structural and interpersonal racism could create power differentials that establish a hierarchy in which the patient is automatically placed into a status of power and dental students are placed in a relative subordinate group [3]. This highlights how damaging structural racism could be, even if professionalism and its principles and standards are not rooted in racist intentions. It also poses a question about how the language used in the training in professionalism could fuel inaction and underreporting in dental students.

This qualitative study also revealed an inequity of power and potential structural racism that exists between tutors and students, and students' concerns about potentially tarnishing working relationships or even their future academic progress if they reacted to tutors. This highlights how structural racism could manifest in dental schools exhibited by tutors who failed to respond when the participants had the courage to report interpersonal racism or unacceptable behaviors, leaving the participants feeling despondent and powerless. The power imbalance that exists between faculty and students is inherent in higher education environments. The structures and policies that exist in dental school could be a barrier, hindering meaningful response and action in the face of racist discourse. Faculty must realize their institutional duty in improving and facilitating dialogue and dismantling damaging power dynamics. Recent studies have highlighted the lack of discourse about power in dentistry [40]. This qualitative study emphasizes the need to further explore how power operates within the context of a dental school.

Our study also revealed unique experiences of interpersonal racism influenced by contemporary issues that the participants faced during the COVID‐19 pandemic. The stigmatization of Asian Chinese participants mirrors previous studies that reported xenophobia against international students [41]. The anonymity afforded by Virtual Learning Environments (VLEs) enabled discriminatory behaviors and inappropriate racist and homophobic comments during online learning sessions. A qualitative study of students' experiences of racism in VLEs also revealed what the author termed as “masked” racism [42]. This echoes existing research that has explored how social media and the internet platforms have allowed anonymous users to express racist beliefs without fear of the consequences [43]. The participants' perceptions about the lack of cultural awareness about PPE is particularly concerning given the findings from a recent survey of UK medical students and newly qualified doctors that found that Black, Asian and Minority Ethnic students were less likely to report receiving sufficient information and training about PPE compared to White students [44]. These three descriptions of interpersonal racism are also illustrations of how institutional racism can manifest, where institutions allow discrimination and racism to go unchallenged because of anonymity or perceived ignorance.

This study had limitations that are common to all qualitative studies. One should be cautious about transferring these findings to other dental settings and should view the findings within their own local context. However, considering that this study was carried out at an ethnically diverse and multicultural dental school treating an equally diverse patient population, it could be an underrepresentation of the experiences of students at less diverse dental schools.

Despite these shortcomings, this study demonstrates clear opportunities for anti‐racism actions in dental education [45]. The observed spectrum of racism highlights the urgent need to train, educate, and equip students and tutors to recognize the full spectrum of racism in dental school and not just overt racist behaviors. All students and academic staff need to be taught active bystander skills for others and active ‘standers’ skills for themselves to be able to openly challenge microaggression in a professional manner. Challenging microaggression in a non‐confrontational, calm, and polite way is an essential step in the pathway of reducing interpersonal racism.

The complex power triad that exists between students, tutors, and patients emphasizes the need to redress this power balance by embracing and encouraging the student voice. This could involve students and academic staff collaborating to develop synchronous ‘top‐down’ and ‘bottom‐up’ changes and strategies fully supported by the leadership of dental schools and teaching hospitals. This will make conversations around racism proactive, rather than purely reactive. Examples of strategies could include staff and student training on cultural competence and zero‐tolerance policies on gender and racial discrimination and intersectional discrimination. These zero‐tolerance policies could be provided to patients alongside their appointments and reiterated again by appropriate signposting in waiting areas. Creating safe spaces for students to share their experiences of covert forms of interpersonal racism and microaggression could empower students, provide psychological support, and facilitate better reporting. These “counter‐spaces” could offer a place of sanctuary to students to unpack their experiences of microaggression, receive validation of their encounters and explore ways to manage the situations with trained student support advisors [46].

Online learning is likely to persist post‐pandemic, which means that this could lead to an increase in “masked” racism, which emphasizes the urgent need to address discrimination on VLEs. We need more research to understand the extent of the problem of VLEs used as a platform for racist discourse in dental schools. Every university using VLEs should provide information on expected online etiquette and behaviors and tackle unacceptable behaviors.

This study highlighted interpersonal, structural, and institutional racism observed on numerous micro and macro‐levels in the dental school. As such, any introduced anti‐racism action must be multi‐faceted to confront racism at every level. We need to recognize that the three levels of racism are not mutually exclusive but are intersecting and can be a direct manifestation of each other. Encouraging change at the interpersonal level without also addressing the overarching structural and institutional practices that enable such behaviors will be futile. Therefore, institutions must thoroughly interrogate the ways in which their culture and policies contribute to racism and the role they play in fuelling systemic racism to impart sustainable and meaningful change.

## CONCLUSIONS

Examining the participants' experiences, reactions, and responses highlighted a cycle in which students are likely to under‐recognize and under‐report racist incidents, resulting in racist attitudes and behaviors remaining unchallenged, with the potential to propagate. This study highlighted the urgent need for further research into dental students' experiences of racism in dentistry. A lack of awareness presents a blind spot for students and faculty. If an individual is unable to recognize and identify racist discourse, how will they know how to tackle it? Precedent sets perceptions, which informs action. Dental schools and educators play an integral role in empowering students and building truly inclusive learning and workspaces, through not only their words but also their actions.

## Supporting information


**APPENDIX** **S1**: Supporting information.
