# Supplementary material for: A student‐led qualitative study to explore dental undergraduates' understanding, experiences, and responses to racism in a dental school
Source: J Public Health Dent. 2022 Jun 21;82(Suppl 1):36–45. doi: 10.1111/jphd.12514 (PMC9328358; doi:10.1111/jphd.12514)
Supplement: Supplementary file 1 — APPENDIX S1: Supporting information. [file JPHD-82-36-s001.docx]

Appendix 1: Hypothetical scenarios included in the topic guide depicting incidents in students, tutors, and patients

| Scenario 1: A dental student is organising an end of term party and has arranged a hog roast for the event. The student decides not to invite Javid, a student in her tutor group because she thinks he will be offended by the hog roast. |
| --- |
| Scenario 2: Clinical tutor A and B have been personal tutors for the past four years. Clinical tutor A finds it difficult to remember the name of the students in his group and asks them to wear name badges when they are on clinic. Clinical tutor B persistently mispronounces the name of the only Black African student in his group and asks if he can call her Kate during a tutorial witnessed by other students. |
| Scenario 3: Two dental students are overheard talking in mocking Indian accents when a South Asian dental student leaves the clinic. |
| Scenario 4  An Asian student is treating a White patient, alongside a dental nurse who is also White. The student leaves the bay to grab some equipment and while absence, the patient remarks in a disgruntled tone “There aren’t many white faces here, are there?” |
